# The Investigation of Shear Fracture Toughness and Structure of ITZ of Limestone Concrete with Different Aggregate Grain Size

**DOI:** 10.3390/ma18173954

**Published:** 2025-08-23

**Authors:** Grzegorz Ludwik Golewski

**Affiliations:** Department of Structural Engineering, Faculty of Civil Engineering and Architecture, Lublin University of Technology, Nadbystrzycka 40 Str., 20-618 Lublin, Poland; g.golewski@pollub.pl; Tel.: +48-81-5384394; Fax: +48-81-5384390

**Keywords:** concrete composite, limestone aggregate, grading, brittleness, fracture toughness, mode II fracture, critical stress-intensity factor (*K*_IIc_), critical unit work of failure (*J*_IIc_), interfacial transition zone (ITZ), microcrack

## Abstract

Due to the shortage of construction aggregates, carbonate rock aggregates—including mainly limestone aggregates—have long been used in structural concrete in many countries worldwide. On the other hand, earlier tests on the shear fracture toughness of concretes with limestone aggregates were very limited and were even abandoned for many years. For the above reasons, in this paper, completely new fracture toughness tests were performed according to the mode II fracture for limestone concretes with different grain size distributions. Two types of aggregate grain were used, i.e., two with maximum grain sizes of 8 mm (M1 series concrete) and 16 mm (M2 series concrete). During the experiments, the critical stress-intensity factor (*K*_IIc_) and critical unit work of failure (*J*_IIc_) were determined. Based on the conducted studies, it was found that higher values of fracture mechanics parameters were noted as the grain sizes of the aggregate used increased. The increases in the analyzed fracture mechanics parameters were noticeably greater in the M2 series concrete compared to the results for the M1 series concrete, specifically by 27% for *K*_IIc_ and 35% for *J*_IIc_. In addition to macroscopic tests, detailed microstructural analyses of the ITZ area between the coarse aggregate grains and the cement matrix were conducted. Based on the captured images, it was determined that, in the M1 series concrete, the contacts between the aggregate grains and the cement paste exhibit a loose structure with visible microcracks. In contrast, the M2 series concrete showed no visible damages within the ITZ area itself nor at their displacement at a distance of approximately a few μm away from this area. This microstructure of both materials resulted in the M1 series concrete being more prone to rapid and sudden fracture propagation, leading to its brittle behavior during the fracture process. In contrast, the large, well-developed limestone aggregate grains in the M2 series concrete facilitated improved stress transfer beyond the ITZ area into the cement matrix, preserving the continuity of the material structure and consequently leading to quasi-plastic behavior of the concrete during the fracture process. The novelty and utilitarianism of the research undertaken result from the fact that exploring the properties of concretes with limestone aggregates using mode II fracture is an important aspect of evaluating the durability and safety of concrete structures subjected mainly to shear forces.

## 1. Introduction

Cementitious concrete has been a basic construction material for many years, used both in building and industrial construction and in road infrastructure [1,2,3]. On the other hand, in terms of the optimal selection of concrete mix ingredients, the interests of concrete technologists are mainly focused on the following two issues [4,5,6]:

- Designing an appropriately durable cement matrix;
- Selecting the most optimal aggregate composition.

Therefore, in addition to the structure of the cement paste, it is the type and geometric properties of the coarse aggregate that determine the quality of the concrete mix and the parameters of the hardened concrete to the greatest extent [7,8,9]. However, it should be noted that the quality of concrete also includes its durability. This important feature of the composite is closely related not only to aggregate gradation but also to its physical and chemical properties [10,11]. Since aggregate constitutes approximately 80% of the weight of all components in concrete, it can influence many very important properties of this building material, such as strength, shrinkage, watertightness, and frost resistance [12,13]. It may also determine the processes of formation and development of damage in the contact layers of the aggregate and the cement matrix [14,15,16]. It should be noted that the interfacial transition zone (ITZ) between the grains of coarse aggregate and cement paste is the primary place where the first defects and microcracks in the concrete composite structure occur [10,11,12,13,14,15,16,17]. However, as proven by previous studies, the susceptibility to damage in the ITZ of materials with brittle cement matrices can be minimized both by appropriate modification of the cement binder [18,19,20,21] and the appropriate selection of the type and gradation of the coarse aggregate [22,23].

Unfortunately, due to the rapidly increasing rate of concrete production, which in recent years has already increased to approximately 10 billion tons (approx. 14 billion m^3^) per year, the resources of coarse aggregates with good strength parameters are also gradually being depleted [24,25]. There is an increasing consumption of both good quality pebble gravel aggregates and very durable crushed stone aggregates obtained from igneous rocks such as basalt and granites [26,27]. It is estimated that the annual global consumption of aggregates is already over 40 billion tons [28,29].

As a result of the continuously increasing demand for conventional aggregates in the construction industry, combined with the rising costs of their extraction and transport, recent years have brought attention to the use of alternative aggregates that had not been previously considered for engineering structures. These include, among other materials, carbonate aggregates, especially limestone aggregates [30,31].

It should be noted that carbonate rock aggregates, due to the aforementioned shortage of construction aggregates, have long been used in structural concrete in many countries worldwide. For example, in certain regions of France [32], as well as in the United States [33] and Japan [34,35], this material has served as one of the basic fillers in structural concrete for many decades. Moreover, both new aggregates obtained from limestone rocks and recycled limestone aggregates are currently used in building structures [36,37,38,39,40].

Limestones consist almost entirely of calcium carbonate, with compressive strengths ranging from 10 to 200 MPa. Thus, these rocks range from very soft to relatively hard; the hardness of limestone on the Mohs scale reaches up to 3 [41]. Therefore, these rocks can be utilized to produce aggregates for both construction and road purposes with varying parameters [42,43].

The advantages of this type of fillers in relation to both ordinary concretes, light concretes, self-compacting concretes, and road pavements have been described in previous studies, such as [33,44,45,46,47,48]. In general, the key properties and characteristics of limestone aggregates that positively affect the microstructure of both the cement matrix and the ITZ between the aggregate and cement paste are the following:

- Well-developed surface of aggregate grains;
- Grain texture;
- Low porosity of aggregates;
- Carbonate dust content.

The aforementioned properties of carbonate aggregates significantly influence the achievement of favorable concrete parameters and enhance their durability under service conditions. Previous studies have shown, among other findings, the positive impact of the share of limestone aggregates in the composition of the concrete mixture in terms of improving the compressive and bending strength factors of hardened concrete and increasing the modulus of elasticity in this type of materials [7,8,49,50]. It has also been found that concretes made on the basis of these aggregates exhibit reduced shrinkage compared to gravel aggregate concretes [9,33], as well as a higher brittleness index [33,44,51].

Other important advantages of rocks used to produce limestone aggregates, such as low water absorption, good frost resistance, and resistance to polishing and surface abrasion, have been thoroughly described in [52]. This publication also characterizes the role of the chemical activity of these fillers in forming a compact ITZ between the grains of coarse aggregate grains and cement paste [53,54]. Particular attention was given to the presence of special diffusion zones within the structure of bonds between the aggregates and cement paste. These unique features, observed exclusively in this type of aggregate, make the ITZ in the area of limestone fillers more strongly bonded to their surface and less susceptible to the formation of decohesive microcracks [52,54,55,56,57].

The referenced article [52] also presents the results of bending fracture toughness tests conducted on ordinary concretes made with limestone aggregates of various gradations. The mechanical tests were complemented by the microscopic evaluation of the contact zones of aggregate grains in concretes with a maximum diameter of coarse aggregate grains up to 8 and 16 mm. Based on these experiments, it was determined that concretes made using limestone aggregate with coarser grains exhibited greater resistance to mode I fracture. These findings have also been confirmed by the results of other studies in this field presented in [57,58,59]. Additionally, optical microscope images showed that, at 100 times magnification of the image, the structure in the area of grains of larger limestone aggregates was compact and free of noticeable damage. However, in the case of concretes made on aggregates with smaller grading, decohesion cracks and loosening were observed at the grain boundary of the coarse aggregate and the cement matrix. Moreover, the structure of the coarse aggregate interfaces in the case of both types of concrete had a direct impact on the results of fracture toughness of these materials. It was observed that both analyzed parameters of fracture mechanics, i.e., critical stress intensity factor $K_{Ic}^{S}$ and critical crack tip opening displacement *CTOD*c, had higher values by 20 to 30% in the case of concretes made on aggregates with larger grain sizes. Based on the existing fracture toughness studies, it was thus established that, under Model I fracture, both linear and non-linear fracture mechanics parameters significantly improved in concretes with limestone aggregates when a filler with larger grain size was used.

However, in real concrete structures (e.g., beam near-support zones, slabs, discs, short cantilevers, notched beams, beams loaded with concentrated forces, frame corners, vertical and horizontal slab connections in retaining walls, and other elements exposed to shear), cracks often arise and develop due to complex stress conditions. The cross-sections of these structural elements are subjected to a combination of tensile and shear forces. This results in fractures developing in sliding mode (Model II) or a mixed fracture mode, i.e., Model I + II or even I + II + III [60,61,62]. However, the studies conducted solely in the opening mode (Model I) do not fully reflect the behavior of the material under operational conditions [63,64]. Furthermore, it should be noted that fracture toughness testing under Model II is especially important because it provides information on the behavior of concrete in the cracked state. Such data are essential for assessing the durability and safety of both building structures and road infrastructure facilities [65,66,67,68].

Therefore, besides fracture toughness tests based on traditional experiments, specifically those conducted under mode I fracture (tension at bending) [69,70], it is also important to assess the fracture toughness of the structural material under II (shearing) and, in some cases, III (tearing) fracture models [71,72,73,74].

When reviewing the available literature on the assessment of fracture processes in concretes with carbonate aggregates, it should be noted that, so far, studies on the fracture toughness of such materials have been carried out almost exclusively using the mode I fracture. Previous research studies have involved composites with a wide range of aggregate grain sizes, from 6 to 29 mm. In previous studies, various parameters of both linear and nonlinear fracture mechanics were analyzed, including critical stress intensity factor (*K*_Ic_), fracture energy (*G*f), and critical crack tip opening displacement (*CTOD*c), mainly for ordinary concrete. Based on the literature review, Table 1 provides a detailed summary of previous studies examining the fracture toughness tests of concrete containing carbonate aggregates (limestone or dolomite).

Furthermore, a review of the available literature on the assessment of fracture processes in limestone materials or concretes containing limestone powders indicates that, so far, the following aspects have also been investigated:

- Fracture toughness, under static and dynamic loads, of limestone rocks from which aggregate for the production of concrete is obtained [80,81];
- The impact of limestone powder content on fracture processes in concrete [82];
- Fracture processes in limestone rocks in first, second, and mixed fracture models using the digital image correlation (DIC) technique [83];
- Fracture processes in limestone rocks using numerical simulations [84];
- Microstructure and morphology of ITZ between coarse aggregates and the paste in concretes with dolomite aggregates [60];
- Relationships between fractal dimension and roughness of the fracture surface after crack propagation in concretes based on crushed limestone aggregates [85,86];
- The effect of limestone powder content on the fracture toughness and fracture processes of cementitious composites reinforced with short polyethylene fibers [87].

Both a detailed analysis of the data presented in Table 1 and a review of other existing studies in this field indicate that research on the fracture toughness of concretes with limestone aggregates in the mode II fracture has so far been very limited or practically neglected. It suffices to say that the only work on this subject encountered in the literature, summarized in the last position of Table 1, was published over 30 years ago (Table 1) [79].

However, the assessment of a material’s susceptibility to damage due to shear stresses is very important. Cracks of this kind occurring in the material can lead to both the accelerated destruction of concrete and corrosion of the concrete reinforcement, especially when shear forces and the migration of liquids into the material structure are combined. Moreover, knowledge of concrete toughness in the second fracture model allows us to better design concrete with increased resistance to fatigue crack propagation, thereby extending the service life of structures. Knowledge of the damage development process within the ITZ of coarse aggregate and cement paste makes it possible to identify where the weakest points in the material occur and to determine the extent to which modifying the composite structure can strengthen them. Such knowledge may be helpful to obtain a construction material with the following attributes:

- Reduced susceptibility to structural defects;
- Increased fracture toughness;
- Improved durability.

Therefore, in order to fill the evident gap in the literature concerning the fracture toughness assessment of concretes with limestone aggregates, experimental studies were carried out to determine the macroscopic fracture toughness according to the mode II fracture in cementitious concretes [88]. Similar to the previous research [52], the influence of coarse aggregate grading on fracture mechanics parameters was analyzed.

In addition, microstructural analyses of transition zones between the grains of coarse aggregate and cement paste in the analyzed materials were also performed. These studies were undertaken due to the fact that the interactions between the microstructure of the material and its properties are of fundamental importance in the construction materials engineering [89,90,91,92,93]. This is demonstrated by, for example, atomistic simulations using density functional theory (DFT) which can provide more details into the underlying mechanisms of toughness between the individual components of the composite [94,95].

Based on the macroscopic examination of the contact points between limestone grains presented in [16], the study evaluated both the morphology of the aggregate–cement paste interface and the nature of cracking in this composite zone. These examinations were conducted using a scanning electron microscope (SEM) at very high magnifications.

In the course of the experiments conducted, the following fundamental parameters of fracture mechanics were determined in both linear and non-linear terms:

- Critical stress intensity factor, *K*_IIc_;
- Critical unit work of failure, *J*_IIc_.

The influence of limestone aggregate grain size on the values of the basic strength parameters of the tested composites was also analyzed. In the course of the experiments, the following parameters were evaluated:

- Compressive strength, *f*_cm_;
- Splitting tensile strength, *f*_ctm_.

The results of the strength tests also made it possible to estimate the brittleness index of the concretes examined [54]. This parameter, defined as the ratio of tensile strength to compressive strength, was particularly important for evaluating the effect of the maximum grain size of limestone aggregates on the behavior of the analyzed composites during the process of their fracture [96,97,98].

Since the grain size of coarse aggregate has a significant impact on the obtained values of concrete fracture mechanics parameters, our research assessed the fracture toughness of composites made on limestone aggregates with significantly different maximum grain sizes. As in the case of previous fracture toughness studies carried out with the mode I fracture [53], two series of structural concretes were analyzed. In order to determine the impact of coarse aggregate grain size on the test results, aggregate mixes with a maximum grain diameter of up to 8 mm (M1 series concrete) and 16 mm (M2 series concrete) were used.

## 2. Experimental Program

### 2.1. Raw Materials

In order to make concrete mixtures, ordinary Portland cement (OPC) produced by Chełm Cement Plant (Chełm, Poland) was used. The binder used in the studies was 32.5 R class and conforms to EN 197-1:2011 standard [99]. The chemical and mineral composition of the OPC is summarized in Table 2. It should be noted that the oxide compositions of the binder were determined by XRF analysis, whereas the mineralogical composition was determined based on the Bogue method. On the other hand, the physical properties of the binder in question are given in Table 3.

**Figure 1:**
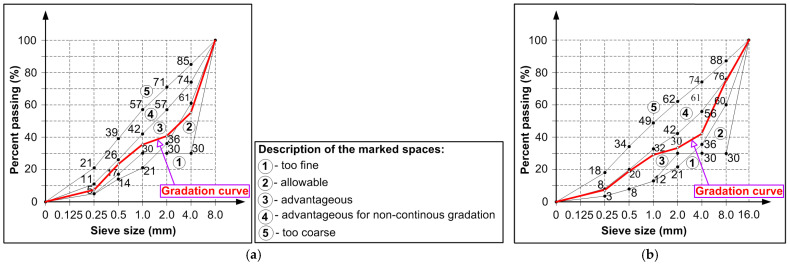
The gradation curve for mixed fine and coarse aggregates: (**a**) of up to 8 mm, (**b**) of up to 16 mm.

Crushed limestone from the Trzuskawica quarry rock bed, with sizes of 2–8 mm (L1) and 8–16 mm (L2), is used as coarse aggregate. Pit sand (S) from the Markuszów deposit is used as fine aggregate with a particle size of 0–2 mm. The significant properties of both aggregates used are given in Table 4, whereas the gradation curves for coarse aggregates with a maximum grain size of up to 8 and 16 mm are shown in Figure 1. It should be noted here that the optimal selection of the aggregate composition for both types of aggregate was determined in accordance with the precise guidelines of the German standard DIN 4226-1 [100].

All aggregate parameters listed in Table 4 were obtained from the information provided by their suppliers. In the case of limestone aggregates, the compressive strength of both types of aggregate referred to the strength of the rocks from which they were extracted. This strength was determined on cubic samples with a side length of 150 mm, taken as drill cores, and then properly prepared and polished. These tests were carried out in accordance with the provisions of EN 13791:2019-12 [101].

In addition to solid components, liquids were also used in the preparation of concrete mixtures. In the study, tap water (W) and the admixture were used. It should be added that tap water met the requirements of standard provision EN 1008:2002 [102], whereas the admixture used to improve the workability of the concrete mixtures was a calcium lignosulfonate-based plasticizer (P), Basf Liquol BV-18 (Ludwigshafen, Germany). It was added at 0.6% of mass of the OPC.

### 2.2. Concrete Mixtures

Two concrete mix designs were evaluated in this study based on limestone aggregates. The experimental variable included the nominal maximum aggregate sizes of coarse aggregates, L1 and L2. For each of the two types of concrete mixtures, two mix designs were evaluated, one with up to 8 mm coarse aggregate, marked as M1, and the other one was made with up to 16 mm aggregate size, marked as M2. The concrete mixture proportions are summarized in Table 5. All concrete mix designs were made with a water-to-cement ratio of 0.4 and the consistency of the concrete mixture V2. The times were measured with the VeBe apparatus in accordance with EN 206-1 [103], and the sand points for the individual mixtures are summarized in Table 5.

### 2.3. Specimens Preparations

#### 2.3.1. Specimens for Testing the Mechanical Parameters of Concretes

Six specimens were made for each of the three types of macroscopic tests, which are described in detail in Section 2.4. This number of specimens for each type of experiment was chosen due to the fact that using six specimens for each kind of test is a good balance between limiting costs and obtaining statistically reliable results. The concrete mixture was prepared in a DZB-300 counter-rotating mixer, manufactured by PROMETAL company (Sopot, Belgrade, Serbia), with a capacity of 150 L and power of 1.1 kW. The making the specimens included the following steps.

In a concrete mixer, the fine and coarse aggregates were added first, and they were mixed for 120 s. Subsequently, the OPC was added, and the mixture was mixed for 180 s. However, aggregates were washed with water and surface dried before mixing them with OPC. In the next step, pre-mixed 0.5 water and plasticizer were mixed for 30 s, and next, this mixture was added to the mixer container and mixed for 90 s. Finally, the remaining amount of water was added, and the all the used concrete mix components were mixed for 120 s. After such a procedure, a homogeneous mass of the mixture was obtained. Next, it was possible to proceed to mold specimens for the various experiments.

For each of the two types of concrete mixtures, cubic specimens of 150 × 150 × 150 mm^3^ were prepared in accordance with the EN 12390-2:2019 standard [104]. The specimens were used for two types of strength tests and fracture toughness tests on the concretes in question. In the case of fracture toughness tests, the cubes contained two initial cracks. The initial cracks in the cubes were formed in the process of their manufacture.

The molding of the samples was performed using two different types of molds. Specimens for strength tests were prepared in plastic molds, while those for fracture toughness tests were cast in stripped steel molds. Prior to casting the specimens, each mold was thoroughly coated with a release agent. Next, the concrete mixture was poured into the molds up to about half their height and initially compacted. In the next step, the molds were filled to the top and compacted again. This two-step method of compacting the concrete mixture in the molds enabled the elimination of excess air voids and resulted in more effective compaction. Each stage of mixture compaction lasted approximately 120 s.

For specimens prepared for fracture toughness tests, special steel inserts with sharpened ends were placed in the molds during this stage of their formation. The inserts were attached to a wooden wedge using screws. Positioning the inserts within the molds aimed to form two initial cracks in the cubes designated for fracture toughness testing under the second fracture mode.

**Figure 2:**
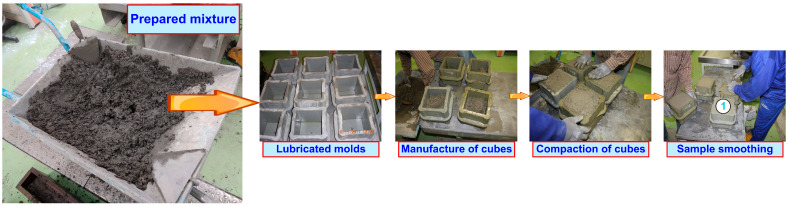
The procedure for molding specimens used for testing the mechanical parameters of concretes: (1) specimen used for the strength tests.

**Figure 3:**
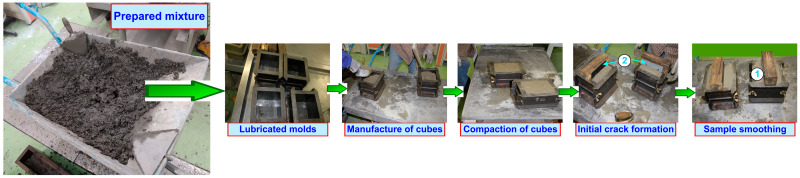
The procedure used for molding specimens used for fracture toughness tests: (1) specimen for the fracture toughness test, (2) steel inserts forming initial cracks.

In the final stage of specimen preparation, both types of specimens were smoothed using steel trowels. This resulted in the samples having almost perfectly smooth top surfaces upon completion. Photos illustrating the specific stages of molding specimens for testing mechanical and fracture toughness parameters are shown in Figure 2 and Figure 3, respectively. An analysis of the photos taken during sample preparation for both types of tests clearly shows that the procedure for forming samples for fracture toughness testing was more complex. It included an additional step involving the creation of initial cracks in the cubes using steel inserts (Figure 3).

After being formed, the specimens were placed on the floor of the same laboratory where they were prepared. Afterwards, the specimens were tightly covered with damp chamois leather cloths, which have good moisture-absorbing properties, and then wrapped with sheets of polyethylene foil. During this period, the samples were intensively poured with water, approximately every 2–3 h. The temperature in the room where the samples were kept was maintained at 20 ± 2 °C. A view of the molded samples after production prepared for strength and fracture toughness tests is presented in Figure 2 and Figure 3.

The specimens pre-matured under these conditions for 2 days. Afterwards, the specimens were de-molded. The specimens for strength tests were de-molded from plastic molds using a compressor. On the other hand, the steel molds used to fabricate the fracture toughness test specimens were de-molded by removing the steel inserts and disassembling the molds.

**Figure 4:**
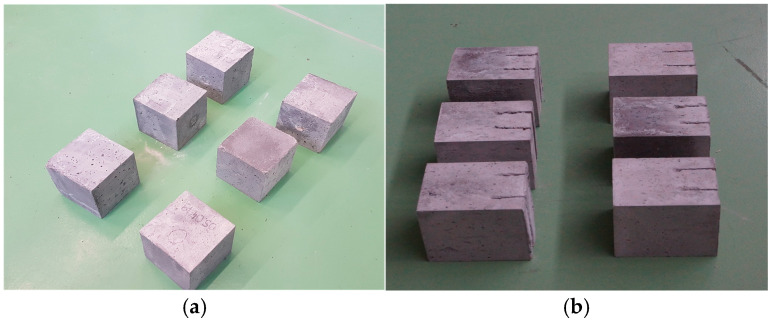
A view of the specimens prepared for strength (**a**) and fracture toughness (**b**) tests.

The de-molded specimens cured under a water curing tank for the first 14 days (t = 20 ± 2 °C, RH = 95–100%). For the next 14 days, the specimens were cured in laboratory conditions (t = 20 ± 2 °C, RH = 40%). Measurements of the strength parameters of the concretes as well as their fracture toughness were carried out after 28 days of manufacturing the specimens. A view of one of the series of specimens prepared for strength and fracture toughness tests after a 28 days of curing is shown in Figure 4.

#### 2.3.2. Specimens for Microstructure Investigation

The fracture surfaces of the samples resulting from the fracture toughness tests conducted according to the mode II fracture were examined under a scanning electron microscope (SEM). The areas located in the immediate vicinity of the initial crack tips in each cube were examined. The samples prepared for microstructural analysis had the following characteristics:

- Shape of the samples: rectangular cubes.
- Sample dimensions: 10 × 10 × 3 mm.
- Number of samples: six samples for each series of concrete.
- Number of photos per sample: thirty photos were taken for each sample, from which the representative photos were selected.
- Sample preparing: the samples before the test were not polished or prepared in any other way, but they were taken as raw.
- Procedures before sample testing: The samples tested in a low vacuum did not require drying and spraying prior to testing. On the other hand, in the case of the samples tested in a high vacuum, the samples were dried for one hour at a temperature of 70 °C and then sprayed with carbon or alloy of gold and palladium in a high-vacuum sputter coater, Q 150 E, manufactured by Angstrom Engineering Inc. (Cambridge, ON, Canada); the thickness of the coated layer was about 50 nm.
- Magnifications used: 20–80,000 times.

**Figure 5:**
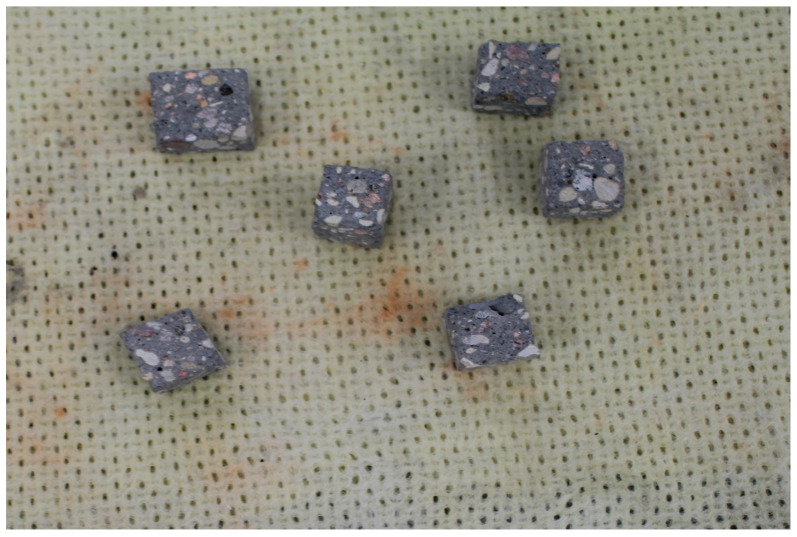
View of the samples prepared for microstructural testing.

Figure 5 presents examples of samples used for microscopic examination. Characteristic grains of limestone aggregates can be observed within the structure of the samples.

### 2.4. Test Program

#### 2.4.1. Compressive Strength

The apparatus employed in the experiment is a microcomputer-controlled pressure testing machine (Walter + Bai ag, type NS19/PA1; Löhningen, Switzerland) with a maximum test force of 3000 kN. The loading rate of between 0.5 MPa/s and 0.8 MPa/s is in accordance with the specifications of the European Standards EN 12390-3: 2011+AC: 2012 [105].

#### 2.4.2. Splitting Tensile Strength

The splitting tensile strength test was performed using the same testing machine that was employed for the compressive strength tests. To ensure the accuracy and repeatability of the results, a very stable load increment was applied during the tests. The loading rate of between 0.04 MPa/s and 0.06 MPa/s is in accordance with the specifications of the European Standards EN 12390-6: 2024-04 [106].

These experimental assumptions were based on the fact that concrete composites are highly sensitive to tensile stresses. In addition, for this type of test, the loading rate is an important factor influencing the results obtained.

#### 2.4.3. Fracture Toughness

Fracture toughness testing using the mode II fracture for concrete with limestone aggregates of varying grain sizes was performed on an MTS 810 testing machine, manufactured by MTS Systems Corp. (Eden Prairie, MN, USA) with the results recorded by computer. The research procedure consisted of static loading of the specimens through a uniform increase in force, controlled by the press head movement speed. The force was applied during the experiments at a rate of 0.25 mm per minute. At this point, it should be noted that the studies of the same test specimens under dynamic loading, in both macroscopic and microscopic terms, were reported in the author’s earlier works [107,108].

**Figure 6:**
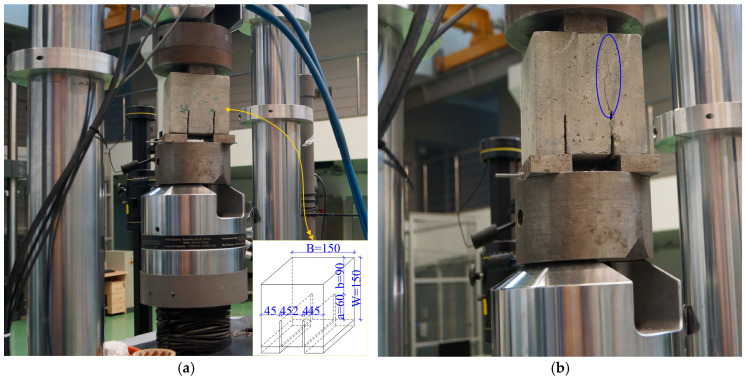
View of the specimen during the mode II fracture examinations: (**a**) during testing, (**b**) at the stage of crack initiation.

A view of the test stand for testing the fracture toughness of concrete according to the mode II fracture and a diagram of the specimen with basic dimensions and markings are shown in Figure 6. This figure, unlike the description of the concrete strength parameter tests, presents two other characteristic photos from the experiment implementation process, namely the following:

- A view of the loaded specimen under the MTS 810 press;
- The moment when the crack appeared in the specimen, highlighted with a blue border, coinciding with the inflection observed in the *F–D* diagram.

During the experiments, the computer connected to the MTS 810 press recorded both the displacement of the lower piston head and the corresponding applied force. Based on these data, it was possible to generate graphs illustrating two relationships, namely:

- Load (*F*)–time (*t*);
- Load–displacement (*D*).

In addition, during the experiments on the MTS 810 press, it was also possible to accurately capture the values of critical forces (*F*_cr_), characterizing the moment of initiation of the first structural crack in the specimens. On the *F–D* graphs, it was identified as a small break or an extremum on the curve. Based on the knowledge of the *F*_cr_, it was possible to calculate the fracture toughness (*K*_IIc_). However, based on the *F–D* curves, it was possible to determine the values of *J*_IIc_.

The fracture toughness *K*_IIc_ of the concretes was determined using the equation provided in Equation (1), derived by J. Watkins [109], shown as follows:

(1) $$K_{IIc}=\frac{5.11F_{cr}}{2Bb}\sqrt{\pi a}$$

However, the critical values of *J*_IIc_ were estimated according to the ASTM E 1820-01 standard [110], based on the following Equation (2):

(2) $$J_{IIc}=\frac{A}{2Bb}.$$

where A is the energy absorbed in the specimen up to the moment of the initial crack growth. This energy can be calculated by finding the area (i.e., by taking the integral) underneath the *F–D* curve to the *F*_cr_ for *b* and *B* according to Figure 6.

Moreover, it should be noted that, in order to avoid errors in the interpretation of the obtained fracture toughness results related to scale effects, J. Watkins carried out additional experimental tests and special numerical simulations. The experimental tests carried out, in which they tested double notched cubic samples (Figure 6), showed that there is a wide range of independence of the geometric size of specimen a/W from compliance function Y(a/W) with respect to the tested stress intensity factor *K*_IIc_ (Figure 6a) [109]. In addition, on the basis of the finite element method, an equation was derived in which stress intensity *K*_IIc_ depends exclusively on the critical value of force *F*_cr_ [109].

#### 2.4.4. Inspection of the ITZ Area

To examine the impact of coarse aggregate grading on the micromorphological properties of concrete structures, SEM QUANTAFEG 250 manufactured in the FEI Company (Hillsboro, OR, USA) was used as observation equipment. The microscope was additionally equipped with energy dispersive spectroscopy EDS EDAX manufactured by Gatan, Inc. (Pleasanton, CA, USA). The type of samples used for the tests and detailed information on the general conditions of the microscopic observations are provided in Section 2.3.2 and are shown in Figure 5.

**Figure 7:**
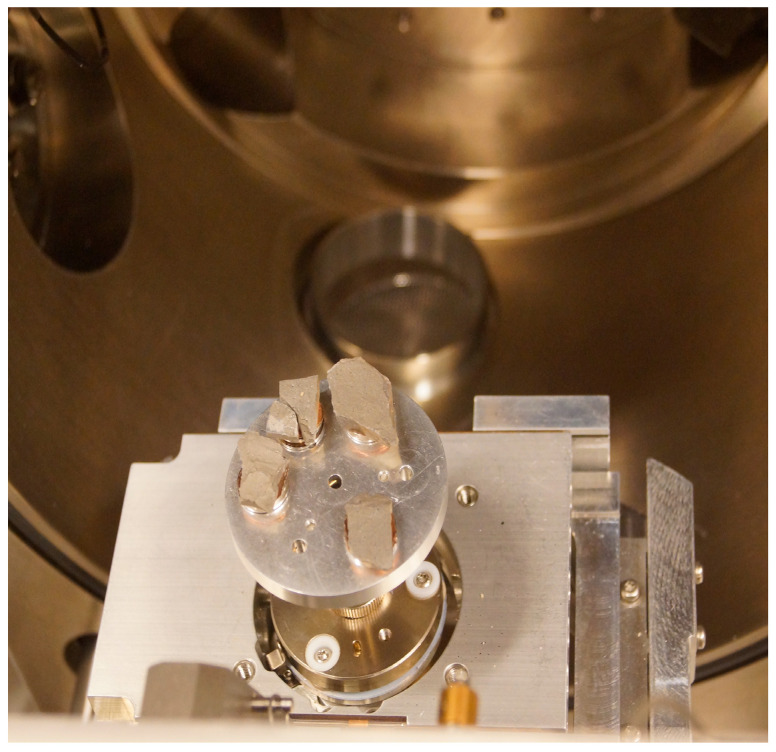
View of samples before the SEM study.

The microstructural analyses focused on the influence of the grain size of coarse limestone aggregate on the morphology of the interface between its grains and the cement paste. During the examination of the ITZ between coarse aggregate grains and the cement paste, the following aspects were analyzed: the contact structure, the bonding strength between the cement matrix and the filler surfaces, the types of phases present in this zone, and their degree of saturation. The extent of defects in this concrete zone was also evaluated. Figure 7 shows representative samples prepared for microscopic examination, positioned on the microscope stage shortly before observation began.

## 3. Experimental Results and Their Evaluation

### 3.1. Impact of Coarse Aggregate Grain Size on Mechanical Parameters, Brittleness, and Fracture Toughness

**Figure 8:**
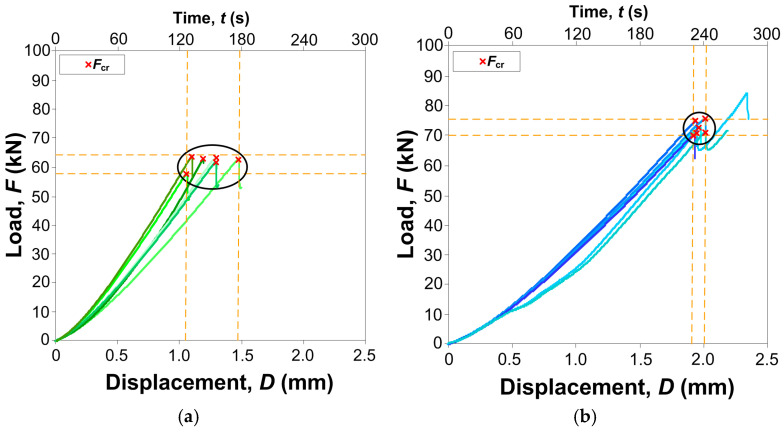
*F–D* curves for all investigated specimens: (**a**) for mix M1 series, (**b**) for mix M2 series.

Table 6 presents the values of the mechanical parameters obtained from the conducted experiments. Additionally, this table contains the calculated brittleness indexes (*BI*) of both analyzed composites. Knowledge of this specific property was, among other things, useful for the subsequent analysis of the *F–D* destruction curves for each material (see Figure 8). To facilitate a comprehensive analysis of the results for each evaluated parameter, the following information is presented in Table 6:

- Calculated average values;
- Estimated statistical factors, i.e., standard deviation (*δ*) and coefficient of variation (*COV*).

An analysis of the results in Table 6 shows a clear increase in both the basic strength parameters and the fracture mechanics parameters for limestone concretes with a larger maximum grain size of coarse aggregate. However, it should be noted that the fracture toughness indicators tested were more susceptible to the change of the aggregate stockpile. The values for the M2 series concrete increased by 27% and 35% compared to those for the M1 series, with respect to the *K*_IIc_ and *J*_IIc_ parameters, respectively. However, the compressive and tensile splitting strengths of the M2 series concrete were higher than the corresponding values *f*_cm_ and *f*_ctm_ obtained for M1 series concrete by 15 and 18%, respectively (Table 6). The results obtained are, therefore, qualitatively consistent with previous studies of the same composites evaluated for fracture toughness using the mode I fracture [52]. Based on this analysis, it was found that concretes made with limestone aggregate of higher grading exhibited bending fracture mechanics parameters $K_{Ic}^{S}$ and CTODc that were more than 20% higher. This phenomenon is further supported by results of other experiments that investigated how the grain size of both limestone and other coarse aggregates in concrete affects the measured values of fracture mechanics parameters [111,112,113,114,115]. This effect was observed both in the case of ordinary concrete and in that of high-performance concrete [59].

To investigate the fracture mechanisms in both concrete series, a detailed examination of the failure curves for both materials was performed. Figure 8 presents a summary of all the *F–D* relationships obtained from the tests for both concrete series. The plots with results were prepared so that each graph displays, on the lower x-axis, the displacement values of the press piston head and, on the upper x-axis, the duration of the experiment. This arrangement made it possible to quickly read the displacement changes in the specimen under increasing load and to easily track the duration of the entire experiment (Figure 8).

The figure also highlights the critical force values (*F*_cr_) that signify the start of propagation of the initial cracks. To enhance the understanding of the results, the scatter zones have been marked with dashed orange lines on the graphs, while the strict scatter areas are outlined with black borders. These additional graphical markings aided in evaluating the consistency of the obtained results (Figure 8).

Based on the macroscopic evaluation of the fracture processes observed during the experiments and the analysis of the *F–D* curves shown in Figure 8, it can be concluded that a two-stage fracture mechanism was identified during the tests. Some of the specimens cracked at the moment of crack initiation at the tip of the initial crack. In such cases, the critical force typically corresponded to the maximum force. Following this event, the *F–D* curve sharply dropped, as the sample experienced sudden failure and could no longer bear any load. This failure pattern was observed in nearly all samples from the M1 series. Based on this, it can be concluded that the fracture mechanism of concrete containing limestone aggregate with grain sizes up to 8 mm was distinctly brittle. This is further supported by the relatively slender shape of the *F–D* curves and their noticeably lower slope compared to the corresponding curves for M2 series concrete. Analysis of the graphs in Figure 8a estimated that the slope of the *F–D* curves for concrete with grain sizes up to 8 mm ranged from 43° to 52°, with an average of 48° across six samples. The increased brittleness of concretes containing limestone aggregate with grain sizes up to 8 mm is also confirmed by the lower *BI* index value for the M1 series concrete compared to that of the M2 series concrete (Table 6).

Additionally, it was observed that the critical force *F*_cr_ values for concrete with a smaller *D*max were highly variable, ranging from 58 to 64 kN. The displacement values of the samples at the occurrence of the *F*_cr_ forces also varied considerably, ranging from 1.05 to 1.45 mm (Figure 8). It should also be noted that the duration of the experiments for the M1 series samples ranged from just over 2 min to nearly 3 min. In the M1 series samples, the initial crack initiated relatively quickly after the load was applied. Such a violent fracture mechanism of the samples also proves the high brittleness of the material from which they were made.

In contrast, a significantly different fracture mechanism was observed in concrete containing aggregate with larger grain sizes. Specimens from this concrete series exhibited initial damage after a significantly longer time, approximately 4 min, and were still able to sustain the load for some time thereafter. Moreover, for the M2 series concrete, a noticeably smaller slope angle of the *F–D* curves and a pronounced ‘flowing’ effect were observed. Based on the analysis of the graphs in Figure 8b, the slope of the *F–D* curves for concrete with grain sizes up to 16 mm was estimated to range from 32° to 37°, with an average of 35.3° across six samples. This indicates a transition in the fracture mode of the samples from brittle to quasi-plastic. Similar effects, characterized by a significant decrease in the slope angle of the failure curves during fracture toughness testing in the second fracture model for concrete with larger-grain gravel aggregate, were also reported in [57].

An analysis of the graphs in Figure 8b also reveals a fairly good consistency of the experimental results for concrete containing aggregate with a larger *D*max. The *F*_cr_ for M2 series concrete ranged from 70 to 75 kN, with displacements at the time of sample failure occurring within a narrow range from 1.8 to 2.0 mm. Based on this, it can be concluded that, within the mode II fracture, concrete with larger-grained limestone aggregates exhibits greater consistency in the results. This observation is further supported by the distinctly different delineation of the results variability range. For the M1 series concrete, the distribution area of the critical force *F*_cr_ results formed an elliptical shape (Figure 8a). However, the obtained *F*_cr_ values for the M2 series concrete fell within a small circular area (Figure 8b).

Nevertheless, the statistical data for both series of concretes presented in Table 6 indicate a good correlation of results. The *COV* did not exceed 10% in any of the groups. Therefore, it is consistent with the literature data, which state that a typical acceptable level of the *COV* for fracture toughness tests is generally considered to be around 10% or less [116]. Hence, the results obtained can therefore be considered statistically reliable.

To explain the mechanisms influencing the observed fracture behavior under shear loads in both analyzed composites, structural analyses of the contact zones between coarse limestone aggregate grains and the cement matrix were conducted. The results of these inspections, along with their discussion, are presented in the following subsection.

### 3.2. Impact of Coarse Aggregate Grain Size on the ITZ Structure and Mechanism of Microfracture

During the structural analyses of composites containing limestone aggregates, the properties of the ITZ between the filler grains and the cement paste were examined. As outlined in Section 2.4.4, the inspections focused on two key factors that could significantly influence the concrete fracture mechanics parameters that were obtained. During the examination of specially prepared microstructure sections, the following aspects were evaluated:

- The morphology and quality of the contacts between the coarse aggregate and the cement matrix, as well as the arrangement of phases and any micro-damage present in this zone;
- The size and characteristics of defects in the ITZ when cracks pass through the aggregate grains within the concrete structure.

**Figure 9:**
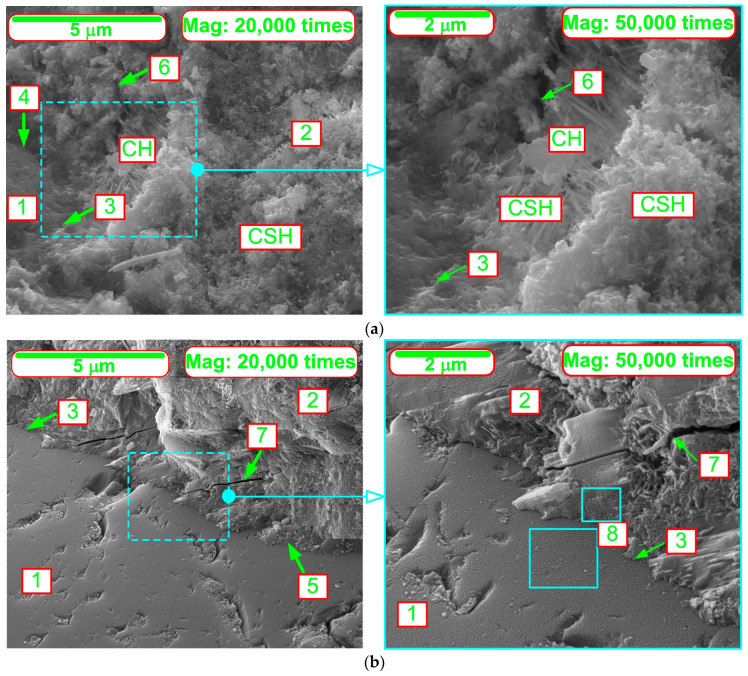
SEM micro-images of the ITZ area structure: (**a**) for mix M1 series, (**b**) for mix M2 series; (1) aggregate, (2) matrix, (3) interface, (4) slightly de-bonded interface, (5) completely dense interface, (6) microcrack, (7) microcrack propagating through the matrix at a certain distance from the ITZ, (8) limestone dust.

**Figure 10:**
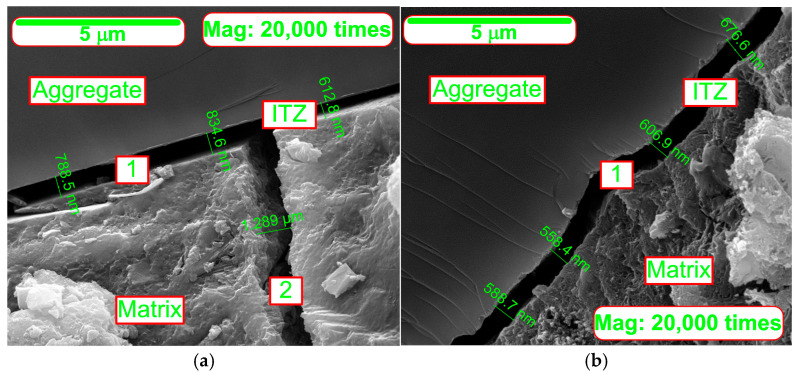
SEM micro-images of the crack system in the ITZ area: (**a**) for mix M1 series, (**b**) for mix M2 series; (1) main crack, (2) secondary crack.

To depict the ITZ structures of both composites as accurately as possible and to identify the differences between them, the analyses were conducted at very high magnifications, namely 20,000×. Moreover, when imaging the contact surface between the coarse aggregate and the cement matrix, additional close-ups of characteristic areas within the ITZ were captured at magnifications of 50,000×. Examples of characteristic ITZ morphology images are presented in Figure 9. Conversely, images illustrating the size and arrangement of cracks at the aggregate–cement paste interface, observed when cleavage-type cracks propagate through the coarse aggregate grains, are presented in Figure 10. Both figures highlight characteristic regions of the concrete composite and identify the phases present in the cement matrix. Additionally, the locations and types of observed damage were indicated.

An analysis of the microscopic images of the ITZ area in Figure 9 indicates that, in the case of concrete with smaller grain sizes, the structure of the cement matrix in this region was highly heterogeneous and poorly compacted. It contained the primary phases of the cement paste, such as calcium hydroxide (CH) and calcium silicate hydrate (CSH), in a relatively loose form. Additionally, microcracks were visible both at the interface between the aggregate and the matrix, as well as within the cement paste structure (Figure 9a). Such defects could undoubtedly reduce the macroscopic fracture toughness of the concrete in this series.

Conversely, in concrete with aggregate grain sizes up to 16 mm, limestone grains exhibiting well-developed surfaces were observed. Moreover, the aggregate structure included both micro-cavities formed during the crushing of rock masses and characteristic fine limestone dust grains present on their surfaces. Limestone dust is also visible both in the aggregate grain area and in the ITZ area (Figure 9b(8)). In general, limestone dust from limestone aggregates can strengthen the ITZ area in concrete, potentially enhancing the overall strength, fracture toughness, and durability of the material. This is achieved by the limestone dust filling voids and refining the microstructure within the ITZ, which is a weaker region between the aggregate and the cement paste. This is confirmed by the results of other studies on this topic [117,118,119,120]. In addition, the limestone dust on the surface of the aggregate grains was chemically reactive (Figure 9b(8)).

These two factors, a well-developed aggregate surface combined with reactive limestone dust, resulted in a strong bond between the matrix and the aggregate in the M2 series concrete, with no obvious defects. The matrix structure in the ITZ was highly compact and exhibited strong adhesion to the grain surface. As a result, microcracks were prevented from reaching the aggregate contact area by the matrix. Thanks to this, the cracks were arrested and diverted some distance away from the grain structure boundary. They developed at a distance of several μm away from the grain boundary within the cement paste structure (Figure 9b). Therefore, the likelihood of defect development was reduced by the compact ITZ in this material. This led to a significant improvement in the fracture toughness of the M2 series composite (Table 6).

During the experiments, the following two primary failure mechanisms were observed: damage initiation in the ITZ area followed by its propagation deep into the material, and the formation of damage within the coarse aggregate grains [85,121]. The phenomenon of damage propagating through aggregate grains was observed primarily in concrete with a larger *D*max. In this case, the cracks within the ITZ were fairly uniform and showed no significant branching. They also exhibited significantly narrower openings compared to the cracks observed in the M1 series concrete (Figure 10b). In contrast, in the composite with a smaller aggregate grain size, more extensive damage and secondary cracks developing perpendicular to the main crack were observed (Figure 10a). This pattern of complex damage resulted in the M1 series concrete exhibiting lower fracture toughness. In turn, increasing the maximum grain size in the concrete mix modified the interface system at the coarse aggregate–cement paste contact, reduced the extent of interfacial cracking, and consequently improved the material fracture toughness.

Clear differences in the ITZ structure of both analyzed materials also affected the changes observed in their fracture processes. The M1 series concrete, characterized by lower grading, exhibited weak ITZs with secondary fractures extending deeply into the cement matrix (Figure 9a and Figure 10a). Consequently, the microstructure of this concrete exhibited reduced fracture toughness and greater susceptibility to rapid, uncontrolled fracture propagation. Therefore, concrete made with limestone aggregate of smaller grain size exhibited significantly greater brittleness during cracking. In turn, the well-developed surface of the limestone aggregate grains in the M2 series concrete, along with a larger diffusion zone, contributed to the continuity of the composite structure. The lack of damage in the ITZ area and its relocation to a certain distance were due to the fact that the larger limestone aggregates acted as stress-dissipating reinforcement. As a result, the shear stresses occurring during the experiments were redistributed beyond the ITZ area (Figure 9b and Figure 10b). This led to a very compact critical zone in the composite, while the fracture processes in the M2 series concrete were quasi-plastic [59].

Factors related to the structure of the ITZ also influenced the consistency of the results obtained from macroscopic tests, affecting both strength resistance and fracture toughness. The higher convergence observed in the mechanical test results for the M2 series concrete resulted from effective contact between the chemically active and well-developed surfaces of the larger aggregate particles. Larger limestone grains reduced the number of crack initiation points in the ITZ zone, thus contributing to a greater homogeneity of the material and limiting the variability of the results obtained from macroscopic tests.

## 4. Discussion

The following article presents original research results assessing the influence of the grain size of coarse limestone aggregates on the basic parameters of the fracture mechanics of ordinary concrete assessed in the mode II fracture.

In fact, experimental research was conducted to evaluate the effect of different types of aggregate compositions commonly used in the concrete industry on the fundamental parameters of concrete fracture mechanics, considering the mode II fracture [49,122,123].

The novelty and utilitarianism of the research undertaken resulted from the fact that exploring the properties of concretes with limestone aggregates using mode II fracture is an important aspect of evaluating the durability and safety of concrete structures subjected mainly to shear forces. Additionally, the experiments evaluated both the linear and nonlinear fracture toughness of two types of concrete composites, featuring coarse aggregate grain sizes of 2–8 mm (M1) and 8–16 mm (M2). The differences in the fracture processes of both materials were also examined. Furthermore, detailed analysis of the contact zones between the aggregate grains and the cement paste in both groups was conducted. The structure of the interface between the two main phases of the composites, as well as the extent of defects in this zone for both materials, was examined.

The research revealed that concretes made with limestone aggregates sized 8 to 16 mm exhibited higher fracture toughness compared to those containing the same type of aggregate sized 2 to 8 mm. The increases in both analyzed fracture mechanics parameters were noticeably greater in the M2 series concrete compared to the results for the M1 series concrete, specifically by the following amounts:

- 27% for *K*_IIc_;
- 35% for *J*_IIc_.

The basic strength parameters were also higher in concrete with limestone aggregate grain sizes up to 16 mm. However, the differences in the results in favor of this material were somewhat smaller compared to the results for concrete with smaller grain sizes. Nevertheless, they increased accordingly by the following:

- 15% for *f*_cm_;
- 18% for *f*_ctm_.

A careful analysis of the macroscopic fracture toughness parameters and fracture mechanisms of both materials revealed that concretes with smaller limestone aggregate grain sizes exhibited distinctly brittle behavior during fracture. In contrast, the concretes of the second series exhibited characteristics of quasi-plastic fracture.

On the other hand, based on the microstructural studies using SEM, it was determined that, in the M1 series concrete, the ITZ between the aggregate grains and the cement paste exhibited a loose structure with visible microcracks. Moreover, besides the main cracks, transverse secondary cracks also developed along the grain boundaries (Figure 9a and Figure 10a). In contrast, the M2 series concrete showed no visible damages within the ITZ area itself nor in its displacement at a distance of approximately a few μm away from this area (Figure 9b and Figure 10b). This microstructure of both materials resulted in the M1 series concrete being more prone to rapid and sudden fracture propagation, leading to its brittle behavior during the fracture process (Figure 8a). In contrast, the large, well-developed limestone aggregate grains in the M2 series concrete facilitated improved stress transfer beyond the ITZ area into the cement matrix, preserving the continuity of the material structure and consequently leading to quasi-plastic behavior of the concrete during the fracture process (Figure 8b).

## 5. Conclusions

Based on the experimental analysis data, the following conclusions were drawn:

1. The grain size of coarse limestone aggregate in ordinary concrete plays a crucial role in affecting both the mechanical properties of the material and the microstructure at the interface between the aggregate grains and the cement paste.
2. Both the basic strength parameters of concretes with limestone aggregate, namely *f*_cm_ and *f*_ctm_, as well as the fracture mechanics parameters evaluated under the mode II fracture, i.e., *K*_IIc_ and *J*_IIc_, exhibit much significantly higher values in concretes made with larger-grained aggregate, with increases ranging from a dozen to over 30% (Table 6).
3. Microstructural tests of the ITZ areas between the coarse aggregate grains and the matrix revealed that the M1 series concrete exhibited weak ITZs, characterized by the presence of secondary cracks propagating into the cement matrix. This microstructure lowered the concrete fracture toughness, making it more vulnerable to rapid and abrupt crack propagation. Therefore, concrete made with smaller-grained limestone aggregate exhibited markedly greater brittleness during fracture.
4. The microstructural study also revealed that the M2 series concrete contained limestone aggregate grains with a well-developed surface and a larger diffusion zone. It can be presumed that larger limestone aggregate grains acted as reinforcement, dissipating stresses within the concrete. This can be evidenced by SEM photos of the interfaces of the coarse aggregate with the cement matrix, which were strongly compact (Figure 9b). As a result, shear stresses were redistributed beyond the ITZ area, which, in this concrete, was homogeneous and densely compacted. Consequently, the fracture processes in the M2 series concrete exhibited a quasi-plastic nature.
5. Testing the properties of concretes with limestone aggregates using the mode II fracture is an important aspect of evaluating the durability and safety of structures that primarily endure shear forces. The results of this type of tests may be particularly useful for determining the load-bearing capacity of the support zone of reinforced concrete beams and other reinforced concrete structures, in which the load-bearing capacity is mainly determined by shear stresses.

## Figures and Tables

**Table 1:** Research of the fracture toughness of concretes containing carbonate aggregates with different particle sizes.

Tested Concrete	Aggregate Size (mm)	Fracture Mode	Test Specimen	Loding Scheme	Analyzed Parameter	Reference
Plain	6, 10, 16	Mode I	beam with one initial crack	4-point bending	*K*_Ic_	[75]
Plain	16	Mode I	beam with one initial crack	3-point bending	*K*_Ic_, *CTOD*c	[18]
Plain	25	Mode I	wedge splitting specimen	splitting	*G*f, *CTOD*c	[76]
Plain	20	Mode I	beam with one initial crack	4-point bending	*K*_Ic_	[59]
Plain	20	Mode I	beam with one initial crack	3-point bending	*G*f	[77]
Plain	29	Mode I	beam with one initial crack	3-point bending	*G*f	[78]
Plain	8, 16	Mode I	beam with one initial crack	3-point bending	*K*_Ic_, *CTOD*c	[52]
Plain	8	Mode I	beam with one initial crack	3-point bending	*K*_Ic_, *CTOD*c	[16]
High strength	20	Mode I	beam with one initial crack	4-point bending	*G*f	[51]
Plain	10	Mode II	cube with two initial cracks	compact shear	*K*_IIc_	[79]

**Table 2:** Chemical and mineralogical composition of the OPC used (% mass).

Oxide	SiO_2_	Al_2_O_3_	CaO	MgO	SO_3_	Fe_2_O_3_	LOI *	C_3_S	C_2_S	C_3_A	C_4_AF
OPC	15.00	2.78	71.06	1.38	4.56	2.72	1.24	60.69	15.82	9.24	7.28
**Phase**	**C_3_S**		**C_2_S**		**C_3_A**		**C_4_AF**		**CaSO_4_ (Gypsum)**		
OPC	60.69		15.82		9.24		7.28		5.10		

* Loss of ignition.

**Table 3:** Physical properties of the OPC used.

Analyzed Parameter	Unit	OPC
Specific Gravity	(g/cm^3^)	3.11
Specific Surface Area	(cm^2^/g)	3300
Average Particle Diameter	(μm)	40.01
Setting Time	(min)	initial—207, final—298
Compressive Strength	(MPa)	after 2 days—23.31, after 28 days—50.02
Appearance	n/a	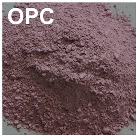

**Table 4:** Properties of fine and coarse aggregates.

Property	Unit	Aggregate Type	
		Fine Aggregate (Sand)	Coarse Aggregate (Limestone)
Specific Density	(g/cm^3^)	2.60	2.85
Bulk Density	(g/cm^3^)	2.20	2.70
Compressive Strength	(MPa)	33.03	100.21
Modulus of Elasticity	(10^2^ MPa)	330	450
Absorption	(%)	0.50	0.32
Appearance	n/a	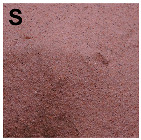	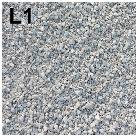 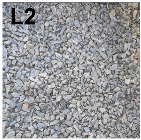

**Table 5:** Concrete mixture proportions used in this study and their properties.

Mix	Constituents of the Concrete Mixtures (kg/m^3^)						Properties of the Concrete Mixtures	
	OPC	W	P	S	L1	L2	Sand Point (%)	VeBe (s)
M1	352	141	2	676	1207	-	40.3	19
M2	352	141	2	676	-	1207	33.3	14

**Table 6:** Average values of analyzed parameters with statistical factors.

**Analyzed Parameter**	**Mix**	
	**M1**	**M2**
$f_{cm}$ (MPa), δ (MPa), COV(%)	39.17, 2.57, 5.7	45.06, 1.09, 2.8
$f_{ctm}$ (MPa), δ (MPa), COV(%)	2.57, 0.2, 6.2	3.03., 0.15, 5.7
$BI=\frac{f_{ctm}}{f_{cm}}$ (–)	6.56	6.72
$K_{IIc}$ (MN/m^3/2^), δ ( MN/m^3/2^), COV(%)	4.26, 0.31, 8.4	5.41, 0.24, 7.6
$J_{IIc}$ (N/m) δ ( N/m), COV(%)	1293.52, 298.61, 9.2	1746.23, 226.72, 8.1

## Data Availability

The original contributions presented in this study are included in the article. Further inquiries can be directed to the corresponding author.
